# Apostoli’s Method of Electrolysis in the Treatment of Fibroid Tumors

**Published:** 1887-09

**Authors:** 


### *APOSTOLI'S METHOD OF ELECTROLYSIS IN THE TREATMENT OF FIBROID TUMORS.*

Dr. F. H. Martin, of Chicago, writes as follows:

While there has been, and for good reasons, a great deal of skepticism in the profession in regard to the value of treatment of fibroid tumors of the uterus by electricity, we are able to discern at present a general tendency to investigate its claims and to take advantage of its results. This tendency in the profession to investigate was brought about by the book of Dr. Apostoli, which appeared in 1884. Now, instead of charlatans monopolizing this valuable therapeutic agent, we find men of position in other countries adopting it—Reiman, of Kiel; Deletong, of Nantes; Hüe, of Rouen; Adolphe Elsassen, of Stuttgart; William Woodham Webb, of London; Gardner, of Montreal; and in this country Englemann and Hulbert, of St. Louis; Bartholow and Massy, of Philadelphia; Baker, of Boston; and Skene and Freeman of Brooklyn.

I wish to confine my remarks this evening to the consideration of Apostoli's method of treatment of fibroid tumors of the uterus. Dr. Apostoli has done away with the mysterious low current. There is a positiveness about his use of electricity that has never been safely imitated by any other method. The reasons for Dr. Apostoli's success rest upon the following facts:

1. The use of strong currents.
2. The adoption af electrodes that make the use of a strong current possible, without harm to innocent tissues and without pain to the patient.
3. The recognition of the peculiar effects of the two poles and the application of them according to requirements.
4. Accurate measurement of current.
5 Rational discrimination in selection of cases.

By the employment of a strong current, short sittings are made practicable, and definite results are obtained. While the amount of electrolytic work done does not depend upon the strength of current, but upon the quantity, definite polar action depends almost exclusively upon the strength of current.

The electrodes used by Apostoli are in all cases of two varieties, the active or internal electrode, and the passive or external electrode. The active internal electrode is for the purpose of concentrating the current at the point where it is most needed. This electrode may be simply a uterine probe of platinum, insulated to the cervical point, or a sharp needle of iridium, insulated to within an inch or two of its point, which will penetrate the mass of the growth from the cervical canal. The passive or external electrode is for the purpose of completing the circuit in such a manner that the strong current shall pass through the largest diameter of the growth, and at the same time diffuse the current sufficiently to prevent pain.

When it passes through the sensitive integument, Apostoli uses for this purpose a biscuit of clay, moulded upon the abdomen, properly connected with one pole of the battery. For this purpose, I use an electrode of my own device. Over the concave surface of a plate of soft metal is stretched an animal membrane, which is attached to the edges in such a manner as to render the interspace between the membrane and the metal water-tight. This space, which is from one-half to one inch in thickness, is filled with warm water. The membraneous surface is applied to the abdomen, and suitable connections made from the metal with one pole of the battery.

The local effect of the negative pole when employed as the active electrode with a strong current is to produce liquefaction of the tumor with which it comes in contact, and is compared to the effect of a caustic alkali. In fact, it is called and is the alkaline pole. This pole, therefore, on account of its effect of rapid solution of tumors, is employed to reduce the size of these abnormal growths.

The local effect of the positive pole when employed as the active electrode, with a strong current, is to produce coagulation and condensation of the tumor with which it comes in contact, and is compared to a caustic acid. It is the acid pole. The coagulating effect of this pole is utilized with marked advantage in controlling the hemorrhage caused by fibroid tumors. Whenever the entire cavity of a bleeding wound can be reached with the positive pole of a sufficiently powerful battery by means of an electrode of platinum, the hemorrhage can surely be checked.

When a current of sufficient strength is employed to obtain the characteristic effect of the electrode, as described above, in order to be of value in the treatment of fibroid tumors, a current of from fifty to one thousand milliampères is necessary. A current of this strength should never be used without proper means of measurement at hand. The strength of the current employed should be varied with the work to be accomplished and the extent of active surface of the internal electrode in contact with tissues to be acted upon.

This scheme of treatment, which I have not been able in the time allotted to more than suggest, is capable of producing rapid and beneficial results. The most distressing hemorrhages can be permanently checked by the coagulating effect of the positive electrode; the most excruciating neuralgic pains, so often accompanying fibroid growths of the uterus, are almost invariably relieved; the smaller tumors are removed entirely, and the large ones rapidly reduced in size by the local effect of the negative pole and the electrolytic and cataphoric action of the current passing through the growth.

The method is free from danger if properly employed. I have yet to see an untoward symptom arise from its use. Its use is accomplished without producing pain enough to require an anæsthetic. The applications are best made in the office.

